# *DeviceD*: Audience–dancer interaction via social media posts and wearable for haptic feedback

**DOI:** 10.1017/wtc.2021.20

**Published:** 2022-02-18

**Authors:** Manoli Moriaty, Lucie Sykes

**Affiliations:** 1 School of Creative and Performing Arts, Liverpool Hope University, Liverpool, United Kingdom; 2 School of Arts, Media and Creative Technology, University of Salford, Salford, United Kingdom

**Keywords:** haptics, performance augmentation, sensors

## Abstract

The performative installation *DeviceD* utilizes a network of systems toward facilitating interaction between dancer, digital media, and audience. Central to the work is a wearable haptic feedback system able to wirelessly deliver vibrotactile stimuli, with the latter initiated by the audience through posting on Twitter social media platform; the system in use searches for specific mentions, hashtags, and keywords, with positive results causing the system to trigger patterns of haptic biofeedback across the wearable’s four actuator motors. The system acts as the intermediator between the audience’s online actions and the dancer receiving physical stimuli; the dancer interprets these biofeedback signals according to Laban’s Effort movement qualities, with the interpretation informing different states of habitual and conscious choreographic performance. In this article, the authors reflect on their collaborative process while developing *DeviceD* alongside a multidisciplinary team of technologists, detailing their experience of refining the technology and methodology behind the work while presenting it in three different settings. A literature review is used to situate the work among contemporary research on interaction over internet and haptics in performance practice; haptic feedback devices have been widely used within artistic work for the past 25 years, with more recent practice and research outputs suggesting an increased interest for haptics in the field of dance research. The authors detail both technological and performative elements making up the work, and provide a transparent evaluation of the system, as means of providing a foundation for further research on wearable haptic devices.

## Introduction

**Figure 1.**
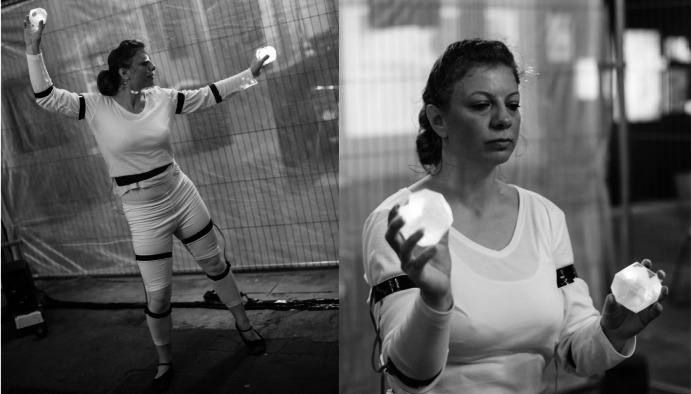
Lucie Sykes performing *DeviceD* during A Grand Exposition at Talbot Mill, November 2017. Sound and visuals are controlled with handheld inertia measurement units, and receive biofeedback signals from the haptic feedback system attached on her limbs and waist. Photograph by Nick Harrison.

The 2017 project *DeviceD* aimed to explore interactive dance through technologies facilitating bidirectional communication between performer and digital information, as well as exploring approaches for remote and local modes of audience participation (for media, see Moriaty 2017). Commissioned and supported by the British Science Association (Liggett et al. 2017), the Manchester Science Festival, and Arts Council England, the work combined interactive music and visuals, motion tracking, haptic feedback, and online audience participation, with its primary aim being the development of a system where the performer’s choreography can be influenced directly through generated data, mirroring the way motion tracking technologies are used toward controlling sound, visuals, lights, and any other type of media that can be affected by digital data (Siegel, 2009). The authors decided on exploring this concept in response to the “general orthodoxy” that interactive performance is based upon, which in the case of music-based interaction follows a model of “gesture → sensor → sound = musical expression” (Salter et al., 2008, p. 249), where “dancers are able to have a direct effect on the music during a performance” (Siegel,^2009^, p. 193). With the choreography generating data able to affect digital media in a multitude of manners, dancers can only utilize the resulting audiovisual material as a way of affecting the choreography through their own hearing and vision. The technology developed for *DeviceD* aimed to overcome this limitation by delivering physical stimuli directly on the dancer’s body through a wearable haptic feedback system (Figure 1). Thus, the improvising body can manipulate the interactive elements of music and visuals in response to haptic feedback, the latter of which being controlled by audience participation through the online social media platform Twitter, with the dancer utilizing Laban’s Effort theory as a means of interpreting the biofeedback inputs.

In this article, the authors describe their collaborative efforts toward creating and performing *DeviceD.* The work is detailed through the utilized concepts, technologies, and methodologies, with particular emphasis on the prototype wearable haptic feedback system created with the assistance of two further collaborators, programmer Barry Carter and music education researcher Dr. Adam Hart. Following a literature review on dance and performance art works exploring online audience interaction, the section “*DeviceD*: System and Performance” contains details on the four technological systems facilitating interaction between dancer, audience, and digital media, as well as describing the feedback loops which emerge for each of the different modes of interaction that are activated during the performance. The section “Performing *DeviceD*” presents the way Laban’s Efforts were utilized toward allowing the dancer to interpret the biofeedback signals. The work is then evaluated in the following section. This is accomplished by describing the challenges met in each of its presentations at three public events, and the actions taken to rectify these. The authors reflect on their experience of performing with the wearable system, and examine recent literature from the field of haptics and interactive dance toward evaluating the work’s technology and performance. The article concludes by suggesting ways for future research of similar systems.

## Internet Interaction in Performance Work

At the time of creating *DeviceD*, Moriaty and Sykes possessed reasonable experience in creating performances featuring real-time interaction between movement and digital media, respectively music and visualizations. Their collaboration in this project aimed to expand on their previous work by incorporating two further elements: online interaction and haptic feedback. This endeavor drew influence from several multimedia works featuring biofeedback and audience participation. The one artist who best embodies such an approach is Stelarc, whose works *Ping Body* and *ParaSite* use systems that allow the performer to experience muscle stimulation according to flows of web traffic:

During the Ping Body performances, what is being considered is a body moving not to the promptings of another body in another place, but rather to Internet activity itself—the body’s proprioception and musculature stimulated not by its internal nervous system but by the external ebb and flow of data (Medien Kunst Netz, 2021).

In *Ping Body*, Stelarc is augmenting his body through not only his infamous “third hand” (Stelarc, 1999), but also via electrodes delivering muscle stimulation to his body (Medien Kunst Netz, 2021). The timing, intensity, and pattern by which these stimuli are delivered are determined according to the actions of remote audiences viewing the performance through the Internet. *ParaSite* employed a similar approach of connecting the performer’s body with web data, albeit this time without input by remotely observing audience members, but rather through randomly selected images which are converted not only into visualizations, but also into the data stimulating the performer’s body through the connected electrodes:

Images gathered from the Internet are mapped onto the body and, driven by a muscle stimulation system, the body becomes a reactive node in an extended virtual nervous system. This system electronically extends the body’s optical and operational parameters beyond its cyborg augmentation of the third arm, muscle randomly scales incoming jpeg images. In real time, the digital data are simultaneously displayed on the body and its immediate environment and, to the characteristics of this data, muscle movement is involuntarily actuated (Studio for Creative Inquiry, n.d.).

At the time when Stelarc created these works (1995–1998), performances featuring interaction via the Internet were rare. In more recent years, with the increase of internet users through the advent of social media, artists have been conceiving new ways of utilizing web traffic toward artistic expression. Dovey’s (2011) *Emotional Stock Market* is a performance art piece where imaginary stockbrokers are “selling” printed sentiments collected from Twitter. The system collecting tweets is searching for specific keywords, which are then categorized and forwarded to three thermal printers. Quite like *ParaSite*, actions of internet users are translated into inputs that the artists utilize for their performance. What is of particular interest here is that the users are unaware of the performance and the ways their online communications are exploited, as opposed to the conscious inputs evident in *Ping Body.* While in the *Emotional Stock Market* user inputs are individually identifiable, other works have been aggregating larger samples of Internet-borne data; the 2018 performance *Dökk* (Abbott, 2019) by the Italian collective Fuse combines dance with motion tracking and interactive sound and visuals. In this work, the performer is able to affect the digital media through the wearable motion tracking system (Perception Neuron), with the nature of the generated audiovisual media determined according to “the sentimental analysis of contents shared of social media” (Fuse, 2018, 2:17). This is achieved through a system searching for specific keywords on Twitter and analyses these according to the “warmth” (Fuse, 2017) exhibited by the social media platform during the performance, with the results ultimately mapped to the intensity of colors in the visuals and “six ghost tracks” (Fuse, 2017) of the work’s soundtrack. As with Stelarc’s *ParaSite*, *Dökk* is extracting data derived from web traffic toward the interactive elements of the performance, without these however being individually identifiable, nor constituting conscious interactions of web users for the performance.

While internet interaction in a live dance performance can be traced as far back as the mid-1990s (Sicchio, 2020), more recent dance works use it to control not only sonic and visual elements, but also as means of affecting dancer’s choreography through wearable systems. Kate Sicchio’s *Feeling Distance* allows audiences viewing the live-streamed performance to “interact with the dancer’s costumes and provide a virtual yet physical touch to the moving bodies they are watching” (Sicchio, 2021). In this work, dancers are wearing costumes with inflatable elements, which “move” in response to audience inputs on a website designed specifically for *Feeling Distance.*

The examined works here provide a context for the concepts that influenced the authors while developing *DeviceD*; utilizing the familiar approach of a dancer interacting with sound and visuals through wearable motion tracking systems, their aims for this work were to allow audiences to interact via online platforms (specifically Twitter) by triggering not only additional sounds and visuals, but also by inducing physical stimuli directly onto the dancer’s body. As it will be explained in more detail in the following section, the focus of the work was not only on developing the necessary technology to facilitate this interaction, but to further explore different modes of audience interaction, with the work exploiting both conscious and unconscious inputs, that is messages specifically posted by audiences observing the performance, as well as tweets that happen to contain the specified keywords.

## *DeviceD*: System and Performance

**Figure 2.**
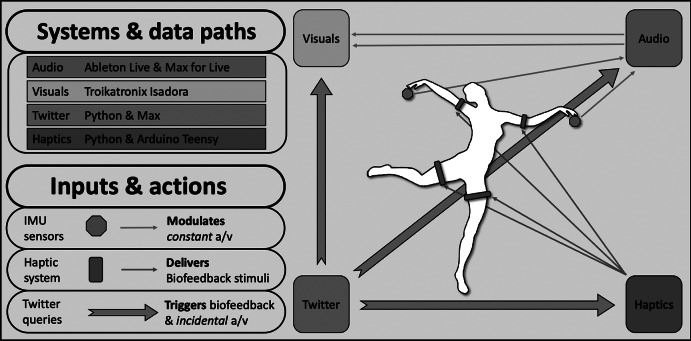
Systems and data paths used for *DeviceD.*

As mentioned earlier, this project aimed to expand on the authors’ previous work on interactive performance works, with each practitioner contributing their experience on designing systems for real-time interaction with sound and visuals via wireless inertia measurement unit (IMU) sensors. These two systems, based on commercially available devices and software, were combined with two further systems developed specifically for *DeviceD*; the software concerned a system able to retrieve information from Twitter and distribute it as OSC messages across the different devices generating the digital media, with the hardware being a wireless wearable designed to deliver haptic stimuli through a set of motor actuators. The four systems and their interconnections are illustrated in Figure 2. This section will firstly provide an overview of the comprising systems and interconnections among these, followed by detailed examinations of each individual system.

The performance creates feedback loops between the dancer and the audience, with the four systems facilitating different modes of interaction. The initial feedback loop concerns the traditional interactive dance approach, were the projected audiovisual material affects the dancer’s choreography, and their resulting movements affect the audiovisual material via the data communicated from the wearable IMUs. This loop is occasionally disrupted by the audience’s Twitter messages, which trigger additional “incidental” audiovisual material, as well as brief biofeedback signals which are delivered through the wearable haptic actuators attached on the dancer’s limbs. The Twitter system makes no distinction between local or remote messages, meaning that while audience present in the performance are able to witness the outcome of their inputs (in the form of a/v material and the dancer’s reaction to the biofeedback signals), Twitter users that happen to send messages containing the targeted queries (hashtags, account mentions, and other keywords) are unconsciously contributing to the performance. As a result, local audiences are engaged in a feedback loop with the dancer, whereas remote audiences exist outside of this loop, as although their Twitter activity is utilized by the performance, they are not aware of the consequences of their actions.

**Figure 3.**
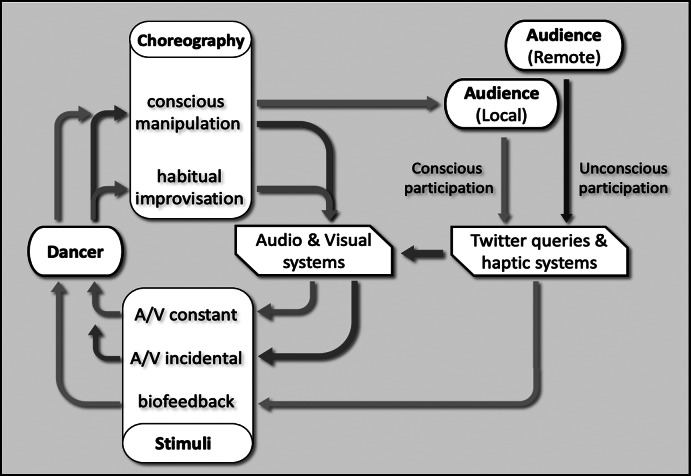
The feedback loops emerging from the interaction between the work’s agents (dancer and audience), stimuli (sound, visuals, and biofeedback), and inputs.

Figure 3 demonstrates the performance’s two distinct feedback loops, one between dancer and system (green) and another between dancer and local audience (blue). On the same figure, local and remote audiences are distinguished (respectively blue and purple), with both inputs resulting in biofeedback signals and “incidental” audiovisual material (*red*), which ultimately result in the choreography changing its state from habitual improvisation to conscious manipulation.

**Figure 4.**
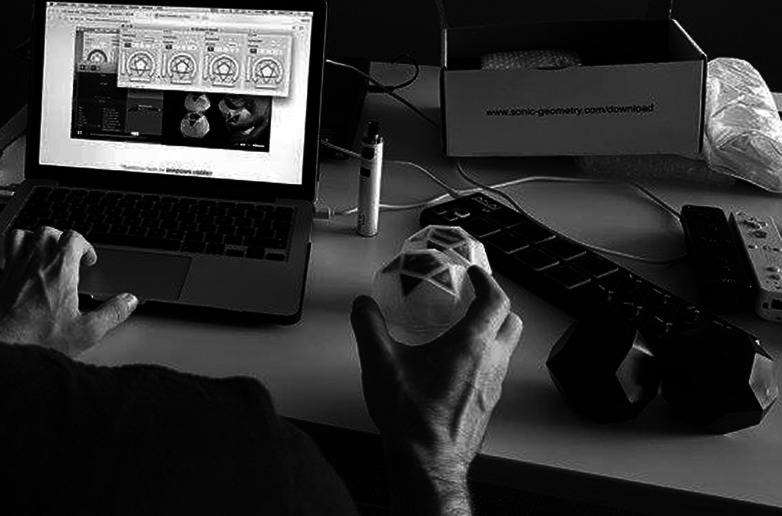
Sonic geometry OTO wireless controllers with inertia measurement unit and touch surfaces.

### Audio System

The sound is generated in Ableton Live, through a combination of synthesis and signal processing. With the work’s presentation format being a durational performance, the sound design is informed by aesthetics of ambient music, consisting of sustained drones with long-form modulations lasting approximately 1 hr. The sonic elements^1^ are arranged in four parts, each containing distinct textures and concentrating on different spectral ranges. This four-part arrangement further accommodated the strategies for sound diffusion and interaction; each of the sonic parts is routed to a distinct nearfield speaker, with the low frequencies reinforced through a subwoofer playing a sum of all four parts. The dancer’s gestural interaction with the sonic elements is facilitated by four Sonic Geometry OTOs,^2^ with the data streams of the contained IMUs mapped to parameters of the devices generating each of the four sonic parts via Ableton’s MIDI mapping matrix. This approach provides the dancer with a choice of controlling different sonic parts during her performance.

In addition to the four-part drones, several fixed sounds (i.e., that experience no further manipulation) are also present, in this case being triggered through the Twitter interaction system (detailed later in this section). These “incidental” samples were designed as to be sonically distinct from the “constant” sonic elements; while the latter generally occupy low-to-mid frequency ranges at modest amplitude levels, the incidental sounds occupy mid-to-high frequency ranges, and are mixed with the composition at significantly higher volumes. Moreover, while the constant sounds possess sustained and slowly evolving morphologies, the samples are of brief duration (average 10s) with rapid changes in their spectromorphological development.

### Visual System

**Figure 5.**
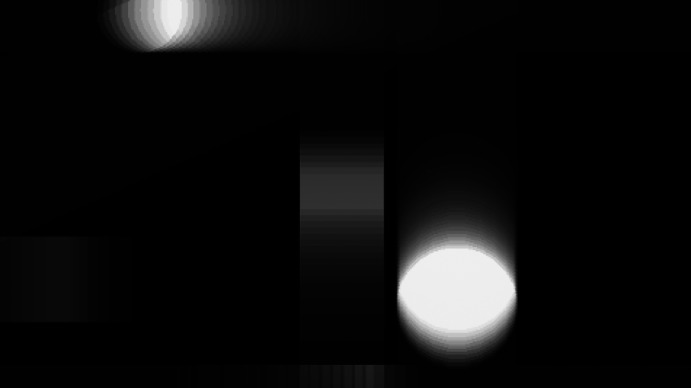
Visuals—geometrical shapes.

**Figure 6.**
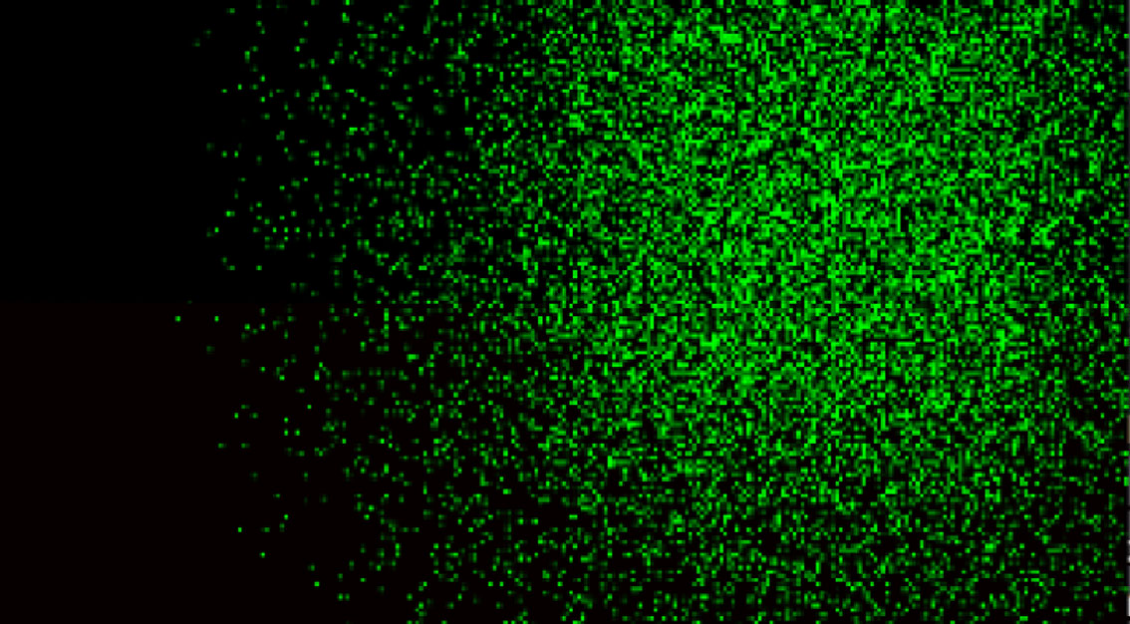
Visuals—particle diffusion.

The visuals are created in the Troikatronix Isadora software; using the drawing functions contained in Isadora’s “shapes” actor module, the visual design comprise of simple geometrical shapes (see Figure 5), partly inspired by Wassily Kandinsky’s aesthetics in his 1925 painting *Yellow–Red–Blue.* ^3^ The shapes are animated in three distinct manners: through Isadora’s “wave generator” actors (similar to Low Frequency Oscillators), by analyzing Ableton’s sonic outputs through the “sound listener” actors, and through the movement data generated by the OTOs’ IMUs. While the latter are initially connected into Ableton for controlling the sonic parameters, the same data streams are simultaneously transmitted into Isadora via Open Sound Control (OSC). As with the strategy in modulating and controlling sound, specific shapes are connected to each OTO, allowing the dancer to move these across the projection space, with the sound analysis of each of the four constant drones affecting the rotation of specific shapes. In addition to the geometrical shapes, modules allowing for visual particles diffusion are used (see Figure 6), with their parameters affected by the sound analysis actors.

**Figure 7.**
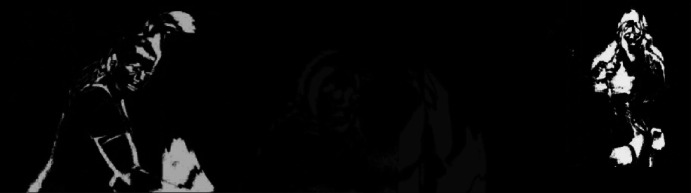
Visuals—three of the “incidental” videos.

As with the distinction between constant and incidental sounds in the audio system, several video recordings are at times projected over the geometrical shapes (see Figure 7). These are triggered through the Twitter interaction system, and while adhering to the overall color scheme, the videos’ organic appearance distinguishes them from the austere geometrical shapes of the constant visuals. As with the incidental sounds, the videos have brief durations, and when projected they appear superimposed over the constant visualizations, albeit slightly transparent.

### Twitter Interaction System

**Figure 8.**
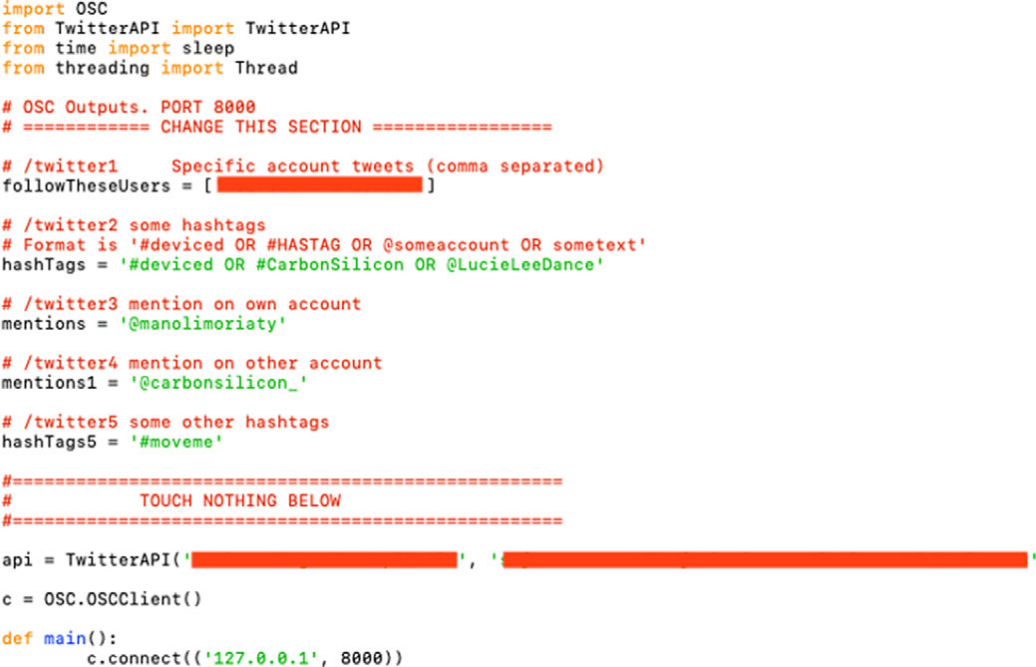
Python script for Twitter search (API key obscured).

**Figure 9.**
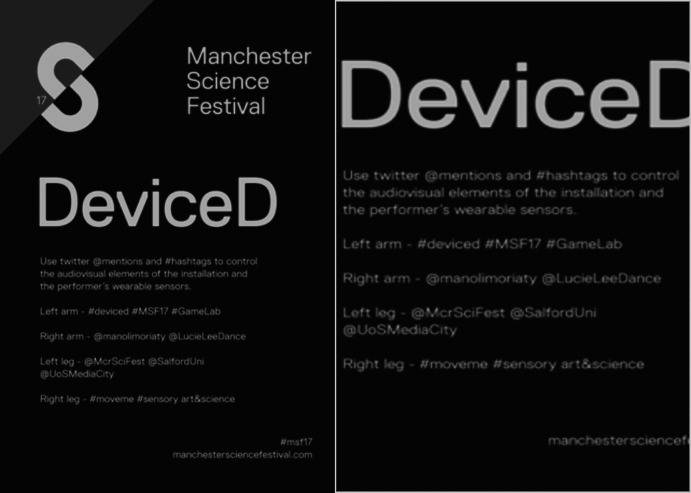
Poster for the Manchester Science Festival performance with the specific terms and corresponding actions.

With the sound and visuals systems created out of commercially available software and hardware, the audience interaction system required the development of a bespoke application. At the time of developing the project, Twitter was chosen as the preferred platform for audience accessibility due to its API key being easily obtainable at that time when compared to other social media platforms, which among other functions, allows developers to perform searches outside Twitter’s own web interface. The Python language was used to create the search system, which allowed the combination of different packages as to first, perform searches on Twitter for different queries (such as account mentions, hashtags, and specific keywords), and second, transmit positive results to other applications via OSC. The Python script (see Figure 8) is structured to search up to five simultaneous queries, with the specific search terms easily modifiable by the user, and output these to a specified OSC port. The easily accessed modification of the search terms was particularly important to implement, as to adjust the system for the different presentation settings. With audiences posting tweets about the event containing specific hashtags and keywords used by each festival (such as the official handle of their Twitter account, or their designated hashtag for the particular event), these terms were inputted into the script as to exploit the data traffic toward the performance as unconscious inputs, or to use a biological metaphor, a commensalistic symbiosis where the audience inputs would “benefit” the artists operating the system while the former remain unaffected (Moriaty, 2020a,b). Once the specific terms were decided upon for each performance, posters were printed and displayed in the performance area, with instructions for the audience on the ways they can interact with the dancer (see Figure 9).

**Figure 10.**
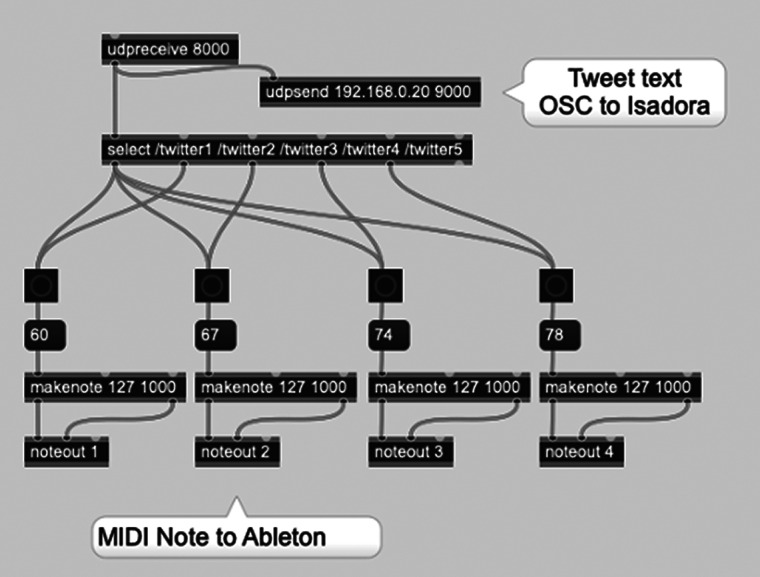
Max patch connecting search results to audio and visual systems.

With the returned search results needing to be communicated to the audio and visual applications, a connecting patch was created in Max (see Figure 10). Using the udpreceive function set at the port specified in the Python script, the incoming results are sorted and distributed in four outputs, with each then converted into MIDI note messages. These are finally sent to Ableton and Isadora, mapped as triggers for the incidental samples and videos respectively, as well as the haptic actuators (described in the next section). In addition, the Max patch also contains a udpsend function routed into Isadora, which allows for the “text read” actor to display the text contained in the tweet retrieved by the search query.

### Haptic System

The final element making up the network of systems used in *DeviceD* is the wearable haptic feedback system, allowing the dancer to experience somatosensory stimuli on her body. The system is comprised of the wearable hardware, a wireless transmitter, and a Python script for OSC data communication between haptic and audio systems.

**Figure 11.**
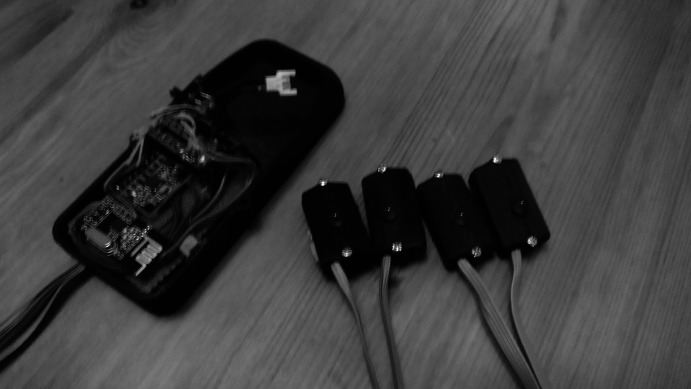
Wearable haptic feedback system, control module (left) and actuators with LEDs (right).

**Figure 12.**
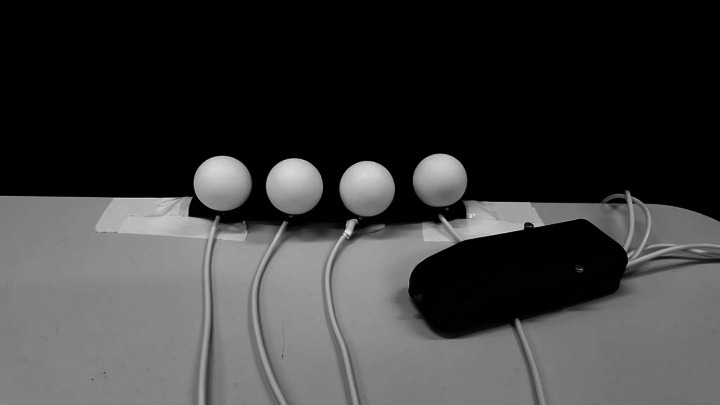
Wearable haptic feedback system, second version with light-diffusion shells.

The wearable was based on the Arduino Teensy LC microcomputer, complemented by the MPU-650 IMU for motion tracking, and the NRF24L01+ wireless module. These components were housed in a printed case, along with the Lipo battery. With this control module attached around the dancer’s waist, the commands are transmitted to four wired modules each housing a motor actuator and a Light Emitting Diode (LED), intended for attachment on the dancer’s limbs (see Figures 11 and 12).

**Figure 13.**
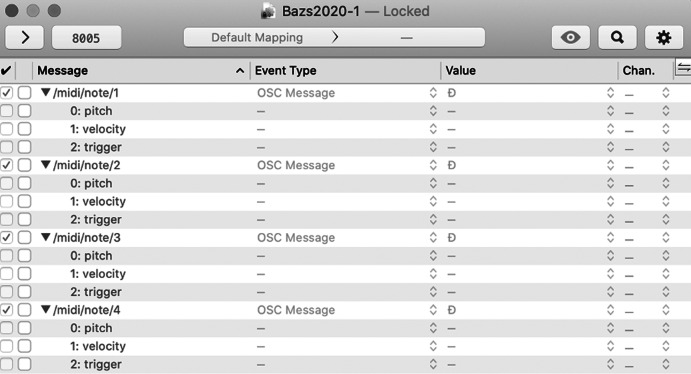
OSCulator patch for OSC data communication between Python script and Ableton.

**Figure 14.**
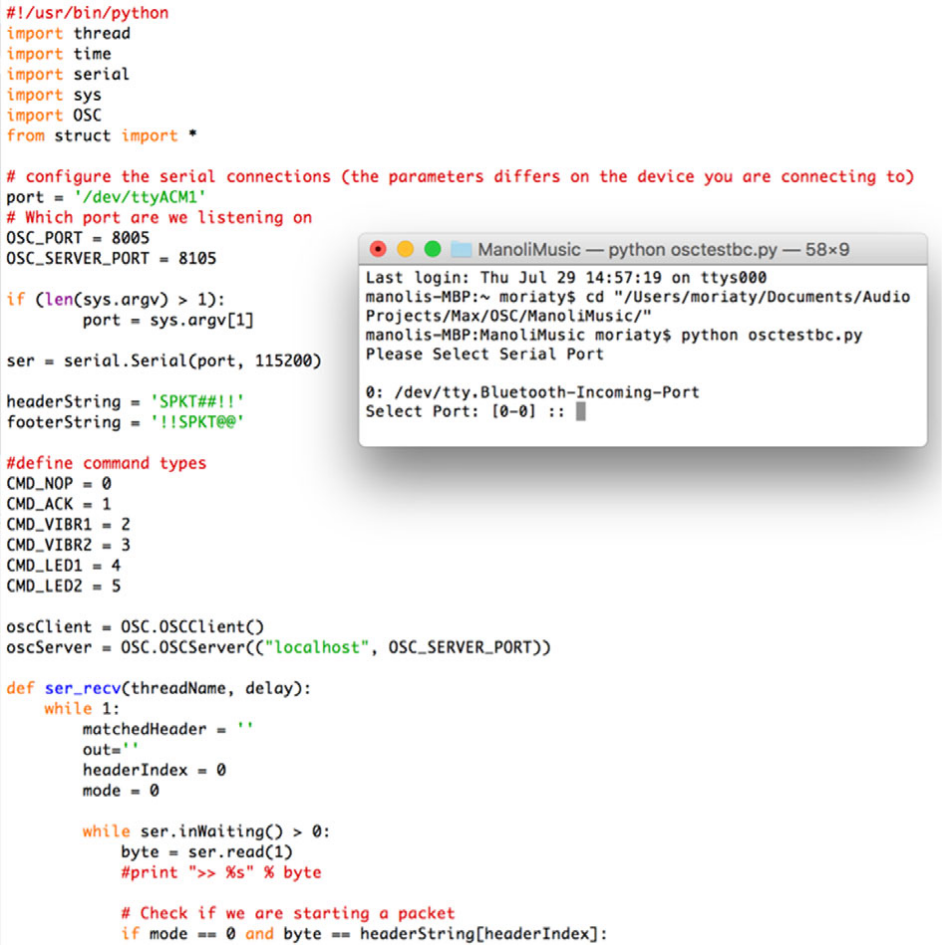
Python script for haptic system and wireless transmitter application.

While the main aim of the wired modules is to deliver biofeedback signals to the dancer, the LEDs were implemented as to provide visual feedback for observers, with the later version of the actuator modules featuring a light diffusion semi-spherical shell as to increase visibility. All hardware modules are attached to the dancer’s body via elastic bands with Velcro fasteners, as to allow for comfort during the performance. The communication of data between control module and computer is achieved through a USB transmitter, which sends information to the former once these have been converted from incoming OSC messages. With the messages originating in Ableton (the Twitter interaction MIDI notes), the conversion into OSC takes place in the OSCulator application (Figure 13), which receives MIDI notes from Ableton and transmits OSC data to the port specified in the control module’s Python script (Figure 14).

## Performing *DeviceD*

This section is the performer’s reflection on the overall experience of performing with the wearable, and interacting with the systems making up *DeviceD.* The performance strategies for the dancer embody improvisation in performance while utilizing concepts of the Laban Movement Analysis notion of effort, which relates to how the body is moving within space. For the improvisational composition created for *DeviceD*, the LMA effort is utilized as means of creating meaningful responses to the biofeedback signals experienced by the dancer from the wearable haptic system.

The Laban Movement Analysis (LMA) is a system of observing, describing the movement and its expressions through four components: *Body*, *Effort*, *Space,* and *Shape* (BESS). Laban’s Effort motion factors are arranged in four components and eight polarities (with *fighting* and *indulging* expressive attitude) as follows: *Space* is *Direct* and *Indirect*, *Time* is *Sudden* and *Sustained*, *Weight* is *Strong* and *Light*, and *Flow* is *Bound* and *Free* (Laban, 1960). Thus, the body moves in between each category and range of polarities with a sense of choice.

For *DeviceD*, sense of choices for each of the four Laban efforts were assigned to distinct haptic inputs, with each of the four actuators designated to a particular effort, as to suggest a change of state to the performer. For example, when the performer felt the vibration on her left arm, the effort quality became *Sustained* as opposed to the right arm with *Sudden* quality. Similarly, a signal on the left leg suggests that the movement quality should become *Strong*, whereas one on the right leg results to *Light.* The whole body moves into these interchangeable Effort qualities throughout the performance.

However, as the whole body moved with the allocated effort qualities for each limb, it was often difficult to connect the performer’s responses to the actual sensations of the vibration and being visible for the observing audiences. Thus, there was a lack of connection between the manipulator/user (the Twitter audience) and the performer’s movement responses to those inputs. Upon reflection, we decided to isolate the limb from the whole body to perform the allocated effort qualities. The shift toward a clear wide range of dynamic expressions was evident when only one limb received the haptic feedback as opposed to the occasions when two or three limbs simultaneously received a signal. During a performance, the performer’s “inner attitudes toward different combinations of the effort elements” (Moore, 2009) activates three motion factors to create Effort Drives, which Laban grouped into *Action*, *Passion*, *Vision*, and *Spell* (Longstaff, 2007). The *Weight*, *Space*, and *Time* Efforts design the Action Drive and enable eight basic effort combinations which are performed with highly crystallized exertions of float, wring, dab, flick, press, slash, glide, and punch. While the body is in a dynamic relationship with the efforts Action Drive can be “transformed by flow” into the variant drives: “Passion Drive (no *Space* or focus), Vision Drive (no *Weight* or physical presence), and Spell Drive (no *Time*, endless), each of these also with eight individual effort combinations” (Longstaff, 2007). Thus, when the haptic feedback was received only on three limbs, it “transmutes” movement qualities through the rhythmical relationships “through intermediary transitional efforts, in which one, two, three or four” (Laban, 1980, p. 185) effort motion factors progressively changed into different Effort Drives. The rhythmical relationship was dependent on the choices of the audience’s Twitter queries. For example, one limb received the vibration, then two, then three, which sent the inner attitude of the performer toward timeless moments of Spell Drive enabling the movements to “radiate a quality of fascination” (Laban, 1980, p. 80).

Furthermore, the Twitter queries and haptic feedback on all four limbs at the same time, shifted the body toward a state of what I would call conscious manipulation: a conscious experience of intentional guidance by the participants (Twitter user). This conscious manipulation activates the variety of Effort Drives in a nonhierarchical order and in random intervals and enables “disruption” from the “habitual” (unintentional reappearance of motion patterns) and other sensory improvisatory responses of the performer. Laban (1960, 1980) suggests that changes in habitual effort patterns can be brought through the “conscious understanding of the structure and rhythm of one’s habitual effort patterns” (p. 105), but he also acknowledges that some are “so ingrained” that it might not be possible to modify them. The interactive possibilities of *DeviceD* offered an interesting investigation into the design of movement material and the improvisatory processes of making creative choreographic decisions toward the “unknown” and an emerging new way of moving. Thus, the improvisatory processes with the Effort qualities were a vital part of “not-quite-known that gives live performance its special brilliance” (Foster, 2003, p. 4).

## Evaluation

While the systems’ operation was overall satisfactory to the extent that the performances were carried out in their entirety, certain challenges emerged during the three public presentations. This section details the main issues faced by the authors during presenting *DeviceD* in three different public events.

The first performance took place at the Oriel Sycharth Gallery as part of the Carbon Meets Silicon II symposium, organized by Wrexham Glyndwr University. During the presentation, the wearable system suffered from poor connectivity with the wireless transmitter, and even though the audiovisual elements were still triggered by Twitter interactions, the haptic actuators were often unresponsive to the same input. This failure had occurred rather infrequently during rehearsals, but it unfortunately presented itself during the actual presentation. This was particularly detrimental for the dancer’s performance; since the biofeedback signals were central to the way the choreography was planned, their failure to trigger was leaving a gap in the dancer’s options for progressing the choreography. At this stage, the dancer resorted to mimicking incoming biofeedback signals whenever the incidental sounds and visuals indicated that a Twitter query was received, as a way of inducing a sense of development in her choreography.

Upon investigation of the different systems, it emerged that the issue focused on a loose component within the wireless transmitter. This was rectified for the following presentation at MediaCityUK during the 2017 edition of the Manchester Science Festival. While the communication between the wearable haptic and the wireless transmitter proved reliable, the Python script managing the communication between wearable and OSCulator developed a server packet delay issue, where incoming messages would become stuck. This caused the biofeedback signals to persist beyond their programmed duration (set at vibrations patterns lasting 10s). In the occasions this issue took place, the dancer had to resort to reaching to the wearable’s central unit (attached around her waist) as to force a manual reset of the unit through the power switch. While this issue emerged rather infrequently, as well as being a distraction for the dancer, it also raised concerns about the reliability of the two Python scripts used in the Twitter and haptic systems.

Ahead of the work’s third presentation, this time at Manchester’s Talbot Mill for the “A Grand Exposition” festival, certain optimizations were implemented in the Python scripts. Focusing primarily on ensuring the stuck messages were eliminated, the code’s reworking resulted in omitting the ability to adjust the actuators’ speed, meaning that the biofeedback stimuli were of fixed intensity, as opposed to the previous varied intensity patterns. In addition, this iteration increased the energy requirements and greatly reduced battery life, something which however did not pose a particular challenge, as each presentation was planned to last approximately 1 hr, which was sufficiently below the duration of a single battery pack.

Some additional findings emerged from the gathered audience feedback; it was noted that audience members were often unaware of the work’s interactive elements, leading to the printing of instructional posters for better clarity. However, due to the interaction necessitating audiences having access to a Twitter account, this meant that participation was exclusive to users of that social media platform. For the final performance at Talbot Mill, a mobile device was available for audiences to interact with the system through the project’s own Twitter account, @devicedeviced.^4^ Another Twitter-borne issue was the limitation posed by the API key which restricted the frequency and volume of search queries the system could perform in close succession, with any queries above the limit resulting in the platform temporarily deactivating the key for 15 minutes. To avoid this issue, the Python script was rewritten as to force the delivered messages to be stored for intervals of 30s. While this alleviated the deactivation issues, it also meant that the effect of incoming tweets was staggered, thus removing a sense of immediacy for the local audience. One solution we considered to tackle this issue was to also provide a tactile MIDI controller mapped to Ableton, which would immediately trigger all three interactive elements, samples, videos, and haptic actuators. However, the authors chose to not implement this during the public presentations, as they deducted that not only would the increased frequency of incoming biofeedback signal would become uncomfortable for the dancer, but more importantly the Internet-based interaction would become redundant, which of course was an element central to the concept behind *DeviceD.*

While highlighting the technological challenges faced by the prototype wearable, it is important to mention the successes of the overall project, and of course acknowledge the significant contributions of our collaborating programmers toward developing the systems while often faced with limited resources, both temporal and material. The dancer found the wearable system to be particularly comfortable to wear while performing due to its low weight relative to the more substantial handheld IMUs, with the wired limb modules often feeling absent while inactive. Although the original prototype’s cable-management was a cause for a limited range of movements, later iterations of the wearable alleviated this issue, allowing the dancer to better focus on the choreographic response to the biofeedback signals.

On reflection, a particularly well-devised element was the concepts driving the interpretation of the biofeedback signals into choreographic structures. This refers to acknowledging that the audience’s interaction cannot be understood as explicit instructions, but rather as arbitrary provocations, with the dancer then given the option to firstly acknowledge the signals, and subsequently include these within her performance. Heather Culbertson, a leading researcher in the field of haptics and robotics, has noted this effect during studies of wearable haptic systems. Identifying different types of somatosensory stimuli into kinaesthetic and tactile modalities, which respectively refer to different types of sensations, such “forces and torques sensed in the muscles, tendons, and joints” and those experienced through “skin mechanoreceptors” such as “pressure, shear, and vibration” (Culbertson et al., 2018). It was not possible to achieve such variety of sensations with the simple operation of the haptic actuators used for *DeviceD.* However, Culbertson notes on applications limited to “binary information using a simple on-off state change” (mirroring the *DeviceD* wearable system’s operation), that appropriate usage can produce useful results, particularly in translating spatial information through vibration delivered on individuals’ skin with the scope of “direct[ing] user[s] to move either away from the cue (repulsive feedback) or toward the cue (attractive feedback)” (Culbertson et al., 2018). While this observation is done in relation to sight-impaired users, such an approach holds useful potential for guiding a dancer. Moreover, of particular interest is the distinction that “the meaning conveyed by haptic icons is often abstract, so the user must learn the meaning behind the icon” (Chan et al., 2005). In relation to the choreographic approach for *DeviceD*, the dancer developed a haptic language toward interpreting the abstract stimuli into comprehensible messages attached to a predetermined outcome that informed her improvisation.

In terms of audience feedback, the presentations were met with overall favorable comments. This was further supported by a sense of anticipation for the dancer’s appearance; since the work’s presentation was ongoing throughout the day of the events while operating as an interactive audiovisual installation, audience members witnessing that state operation were informed about the eventual dance element of the work at certain times, and would often return to experience the full performance. As such, it was determined that while the audience interaction with the digital media was a point of interest, it was the interaction with the dancer that captured visitors’ attention. Comments relating to how the dancer’s movements affected the digital media were less conclusive. This refers to the notion of legibility of interaction, which suggests that the complexity of interactive systems necessitates that external observers are either intimately aware of the systems mappings, or are invited to reach their own reductive conclusions (Salter et al., 2008). Similarly, the authors do not consider the opaque interaction employed for *DeviceD* as a drawback, but rather as means for observers to avoid focusing on the interactivity, but rather on the emerging performative qualities of the work.

## Conclusions and Future Research

This article presented the authors’ collaboration toward developing *DeviceD.* While the work utilized a rudimentary haptic feedback system, the arrangement and wearable design is sufficient to enable dancers to perform both comfortably and with clearly received somatosensory stimuli. The well-known Laban Efforts concept forms the basis for the dancer’s framework, able to interpret abstract biofeedback inputs into both habitual and conscious responses which informed the improvisational composition.

Since the last public presentations of *DeviceD* in 2017, the authors continued their efforts toward expanding the combined systems in new directions. Their most recent workshop^5^ in early 2020 resulted in omitting the external triggers and internet interaction, and reconfigured the system to a more compact form; the haptic actuators and IMU were attached on the dancer’s forearms, with their mapping redesigned as for the haptic stimuli to be generated by both movement and sound analysis, and delivered in the form of variable patterns across the dancer’s upper limbs. This workshop was intended to contribute insights toward a research proposal aiming to expand understanding of the effects and applications of wearable haptic systems on dance performance. With those plans curtailed due to the restrictions brought by the global pandemic, the authors took the opportunity to reflect on their previous work by examining publications on haptics from both artistic and scientific perspectives. Recently, the field’s research community has produced significant technological advancement in sensor and actuator design, as well as the use of telematics and internet-enabled interaction (Mitchel et al. 2021). A further research trend emerged, that of utilizing wearable haptic feedback systems toward sense augmentation for individuals with visual and hearing impairments (Hossny et al., 2015; Abad et al., 2020; McCormick et al., 2020). Considering that similar efforts are currently taking place by dance companies toward including performers and audiences with visual and hearing impairments (Brand, 2019; Watlington, 2021), the authors consider this area to hold potential for future expansion of the findings to emerge from their previous work on *DeviceD.*

## Data Availability

Data sharing is not applicable to this article as no new data were created or analyzed in this study. All code created for this study is available by request from the authors under Creative Commons Attribution 4.0 International License.

## Footnotes

1 The sounds described here can be heard on the project’s webpage, manolimoriaty.com/performance/deviced/

2 OTO is a wireless controller containing IMUs and touch surfaces (see Figure 4). Sonic Geometry’s website is now defunct, with information on their legacy products available at www.instagram.com/sonicgeometry

3 Image of the drawing is available at https://www.wassily-kandinsky.org/Yellow-Red-Blue.jsp

4
https://twitter.com/devicedeviced

5 An annotated video presentation of the workshop outcome is available at https://www.youtube.com/watch?v=o5QfW7IY_hI
